# Facilitators and barriers influencing patient safety in Swedish hospitals: a qualitative study of nurses’ perceptions

**DOI:** 10.1186/1472-6955-13-23

**Published:** 2014-08-13

**Authors:** Mikaela Ridelberg, Kerstin Roback, Per Nilsen

**Affiliations:** 1 Department of Medical and Health Sciences, Division of Health Care Analysis, Linkoping University, Linköping 581 83, Sweden

**Keywords:** Patient safety, Nurse, Qualitative content analysis, Interview, Implementation, Intervention, Multifaceted

## Abstract

**Background:**

Sweden has undertaken many national, regional, and local initiatives to improve patient safety since the mid-2000s, but solid evidence of effectiveness for many solutions is often lacking. Nurses play a vital role in patient safety, constituting 71% of the workforce in Swedish health care. This interview study aimed to explore perceived facilitators and barriers influencing patient safety among nurses involved in the direct provision of care. Considering the importance of nurses with regard to patient safety, this knowledge could facilitate the development and implementation of better solutions.

**Methods:**

A qualitative study with semi-structured individual interviews was carried out. The study population consisted of 12 registered nurses at general hospitals in Sweden. Data were analyzed using qualitative content analysis.

**Results:**

The nurses identified 22 factors that influenced patient safety within seven categories: ‘patient factors’, ‘individual staff factors’, ‘team factors’, ‘task and technology factors’, ‘work environment factors’, ‘organizational and management factors’, and ‘institutional context factors’. Twelve of the 22 factors functioned as both facilitators and barriers, six factors were perceived only as barriers, and four only as facilitators. There were no specific patterns showing that barriers or facilitators were more common in any category.

**Conclusion:**

A broad range of factors are important for patient safety according to registered nurses working in general hospitals in Sweden. The nurses identified facilitators and barriers to improved patient safety at multiple system levels, indicating that complex multifaceted initiatives are required to address patient safety issues. This study encourages further research to achieve a more explicit understanding of the problems and solutions to patient safety.

## Background

The Institute of Medicine report *To Err is Human* in 1999 [1] generated tremendous public and media attention, setting the stage for unprecedented efforts and activities to improve patient safety. Sweden has undertaken many national, regional, and local initiatives to improve patient safety since the mid-2000s, with increased activity over the last few years. A national study from 2008 showed that the incidence (8.6%) of adverse events in Swedish hospitals was not lower than international comparisons [2]. This brought increased focus to patient safety in Swedish health care. The Swedish Association of Local Authorities and Region (SALAR), representing the county councils (health care regions) and municipalities, has played a leading role in efforts for improved patient safety. They have organized patient safety conferences, set up networks of experts and policy-makers and published widely distributed handbooks and evidence-based guidelines for health issues such as falls, pressure ulcers, medication errors in health care transitions and health care-associated infections.

In 2011, a new law on patient safety was introduced and a government-supported financial incentive plan was initiated by SALAR ), which has allocated over 2 billion SEK for 2011–2014 to county councils that carry out certain patient safety-enhancing actions and meet established requirements for specific patient safety indicators. A vision of zero tolerance for adverse events has been discussed in Swedish health care [3,4].

How can the high ambition for improved patient safety in Sweden be achieved? There are few evidence-based patient safety practices that ensure improved patient safety, if implemented with fidelity. Rather, much patient safety work tends to be pragmatic and experience-based instead of relying on solid evidence of effectiveness [5]. Walshe and Boaden (p. 224) [6] complain that many interventions are simply based on “a hunch that they may work” and Vincent [7] (p. 374) believes that the urge to “get on and change things” often takes precedence over carefully planned and evaluated interventions. Thus, it is important to investigate perceived facilitators and barriers to improved patient safety among those who are involved in the direct provision of health care.

Nurses are critical to patient safety. They often have an important role as the coordinator of multidisciplinary care and are involved in many aspects of patient care, from providing comfort and hygiene to administering drugs, updating medical records, as well as handling some therapeutic and diagnostic procedures. Nurse staffing levels, workload, and education levels having been linked to various patient safety outcomes [8-10]. Furthermore, nurses constitute the largest professional group in Swedish health care; 43% of health care professionals are registered nurses and 28% are nurse assistants [11].

Considering the importance of nurses with regard to patient safety, knowledge about their perceptions of the factors that influence patient safety could facilitate the development and implementation of better solutions. It is also possible that their perceptions could help identify less-known patient safety issues. Moreover, solutions based on nurses’ views are likely to be more effective. Based on interviews, the aim of this study was to explore important factors influencing patient safety, as perceived by registered nurses in general hospital care in Sweden.

## Methods

### Study setting

The Swedish health care system is organized at national, regional and local levels. At the national level, the Ministry of Health and Social Affairs establishes policies for health and medical care and sets the primary agenda for health care. At the regional level, the responsibility for financing and provision of health care is decentralized to 21 health care regions (county councils). The county councils have full budgetary responsibility for providing health care to all citizens. At the local level, municipalities are responsible for social care and elderly care.

The Swedish health care system is primarily funded by taxes. In addition to tax revenues, financing of health care services is supplemented by governmental grants and patient fees. According to the Swedish health care act (1982:365), all residents are insured by the state and should have equal access to different health care services [12].

### Study design and participants

In response to the explorative study aims, we undertook a qualitative interview study. We recruited 11 female nurses and 1 male nurse, all with direct patient contact, for individual interviews (details provided in Table 1). All were registered nurses (referred to simply as nurses in the study). Their average age was 48 years (range 27–63 years) and they had worked as nurses for an average of 19 years (range 4–40 years). The nurses came from eight general hospitals in Sweden in six county councils.

**Table 1:** Description of interviewed nurses

**Interviewee number**	**Age**	**Sex**	**Years of working with direct patient care**	**Designated patient safety responsibility**	**Location**
N1	60	F	40	Yes	Urban
N2	46	F	6	No	Urban
N3	60	F	5	Yes	Urban
N4	63	F	40	Yes	Urban
N5	27	F	5	No	Rural
N6	47	F	24	No	Rural
N7	55	F	33	Yes	Rural
N8	50	F	7	Yes	Rural
N9	39	M	11	No	Rural
N10	40	F	20	No	Urban
N11	32	F	4	Did not answer	Rural
N12	60	F	35	No	Urban

The nurses were recruited by means of purposive sampling. Patient safety officers, health care practitioners who hold key positions in the county councils’ patient safety work [13], asked for nurses in their respective county councils who might be interested in discussing their patient safety work. The purposive sampling approach was intended to obtain heterogeneity with regard to demographics, age, and gender, as well as the number of years in practice.

Nurses who agreed to be contacted by the researcher were sent an e-mail with information about the aims of the study, estimated duration of the interview, confidentiality and that participation was voluntary. Nurses who were willing to participate in the study were asked to reply to the e-mail and suggest a time for an interview. Before the interview took place, information about the study was provided again and participants gave consent to participate. They also gave consent to be quoted along with information about their age, sex, how many years of working with direct patient care they had and whether they had designated patient safety responsibility in their work. They were informed that they could withdraw at any time during the interview and that the data would be handled with confidentiality. Ethical approval was obtained from the Regional Ethical Review Board in Linköping.

### Data collection

Semi-structured individual interviews were carried out by the first author who has a background in nursing and clinical patient safety work. The first three interviews were conducted face to face in an office of the respondents’ choice (at their ward or clinic); the other interviews were conducted over the telephone [14]. The face-to-face interviews lasted from 35 to 60 minutes and the telephone interviews between 20 and 45 minutes. The interviews were conducted between June and September 2012 during regular working hours to facilitate participation. All interviews were audio-recorded with the respondents’ permission.

A semi-structured interview guide with open-ended questions was developed by the authors. Each interview started with an open question asking the respondent to describe their patient safety work. The interview was then based on two overarching questions: “What do you believe has been most important to improve patient safety where you work?” and “What do you believe hinders improved patient safety where you work?” We considered patient safety in simple terms of prevention of errors and adverse effects to patients associated with health care [15]. Probing questions were used to obtain more elaborative answers.

### Data analysis

Interviews were transcribed verbatim and then reviewed by the interviewer for accuracy. Data were then entered into Nvivo v 9 for data management. Qualitative content analysis was used in accordance with Hsieh and Shannon [16]. Qualitative content analysis is a technique for analysis of texts based on empirical data with an explorative and descriptive character [17].

All authors read all transcripts to obtain an understanding of the whole. The transcripts were then coded by the first author using conventional content analysis, which entails a structured analysis process to code and categorize the data [16]. The next step was to highlight words in the text that captured various key statements and thoughts in relation to the study aim, that is, factors perceived to influence patient safety [16]. Key statements were given labels to describe their contents [18], which then formed factors that were categorized as (1) facilitators, (2) barriers, or (3) facilitators and barriers. Facilitators were factors that respondents perceived to have a positive impact on patient safety, whereas barriers were factors they perceived to have a negative influence on patient safety. Factors that were identified as both facilitators and barriers acted in a dual sense, positively or negatively affecting patient safety according to the respondents’ different descriptions.

The authors approached the text several times. During this process, codes that reflected more than one key statement or thought developed; the codes were then aggregated into clusters based on similarity of the content and their relation to each other. After re-examination, the initial clusters were merged into categories. The categories were given labels that provided an overall description of their content [16]. The categories were re-examined to ascertain that they were defined so that they were internally as homogeneous as possible and externally as heterogeneous as possible [17].

All authors discussed the content of the categories using triangulating analysis, that is, the authors independently analyzed the same data and compared their findings. Several 2-hour meetings took place to discuss the analysis of factors. In the next step, the categories consisting of facilitators and barriers to patient safety were mapped onto different categories of a multilevel framework developed by Vincent et al. [19]. This framework categorizes various influences on injuries produced by medical care (i.e., factors) into seven categories (i.e., types of factors), providing a conceptual basis for analyzing these injuries and allowing for a wide range of possible influences to be considered [7]. This mapping occurred as we recognized that the emerging coding structure was very similar to Vincent’s framework, which is well recognized in the patient safety field. This deductive approach can use an unconstrained matrix that allows new categories to be created, adhering to principles of inductive analysis [18].

Discussion about this mapping continued until no inconsistencies existed and a shared understanding was reached in order to prevent researcher bias and strengthen the internal validity [20]. Representative quotations were selected to illustrate the findings. Quotations were then translated from Swedish to English by the first author, and then re-examined and re-translated by all authors for accuracy.

## Results

Table 2 presents a summary of the categories and the factors associated with influencing patient safety, as perceived by the nurses.

**Table 2:** Factors influencing patient safety, as perceived by the nurses

**Category**	**Description of the category**	**Barriers (B) and facilitators (F)**
Patient factors	Patient factors relate to patients’ influence on patient safety as perceived by the nurses	Patient interaction (B + F)
		Patient engagement (F)
Individual staff factors	Individual staff factors refer to various personal characteristics of the nurses and other health care providers that the nurses perceived to influence patient safety	Interest and knowledge (F)
		Skills and abilities (B + F)
		Feelings (B)
		Fallibility (B)
Team factors	Team factors refer to various aspects of the interaction between nurses and other health care providers that the nurses perceived to influence patient safety	Collaboration in multiprofessional teams (B + F)
		Communication with colleagues (B + F)
Task and technology factors	Task and technology factors concern workplace technologies and processes involved in storing and sharing of data, information and knowledge that the nurses perceived to influence patient safety	Collecting, storing and sharing patient safety-related data and information (B + F)
		Medical records (B + F)
		Incident reporting (B + F)
		Computerized technology (B + F)
		Written protocols (F)
Work environment factors	Work environment factors relate to workplace conditions that the nurses perceived to influence patient safety	Structures and forums for learning from errors (B + F)
		Work schedule (B)
		Staffing levels and competence mix (B + F)
		Physical environment (B)
Organizational and management factors	Organizational and management factors concern conditions of the health care organization (beyond the specific workplace in which the nurses work) that the nurses perceived to influence patient safety	Leadership (B + F)
		Financial resources (B)
Institutional context factors	Institutional context factors refer to conditions of the outer context of the health care organization that the nurses perceived to influence patient safety	Use of knowledge from external sources (B + F)
		Communication with people external to the workplace (B)
		Societal interest in patient safety (F)

### Patient factors

The nurses believed that patient interaction could facilitate or hinder enhanced patient safety. According to the nurses, patient safety was positively affected by having an open dialogue with patients so that they knew who to contact when something had gone wrong. Well-structured information provided both orally and in writing facilitated patient safety. On the other hand, poor patient interaction due to communication problems could hinder patient safety.

*If they feel unsafe in a situation, it will go wrong. Or if they haven’t really understood what we’ve said, then it will go wrong, and you could call that patient un-safety.* N 9

Some nurses said that patient safety could be positively influenced by patient engagement, that is, when patients were involved in their own care and their next of kin were involved in the care.

*You can make patients aware of patient safety. A small leaflet with information about our work with patient safety, that [we] always check the patient’s identity, just basic stuff, could help. I often think that you must assume that the patient has never been in hospital before, and then an explanation of how we work with patient safety may be good.* N 11

### Individual staff factors

The nurses believed that having a personal interest in patient safety issues had a positive impact on patient safety.

*I have undertaken education in patient safety at [a Swedish university]. It was a course that I requested when I resigned as manager three years ago because I wanted to know a bit more about it.* N 1

Patient safety was also facilitated by several skills and abilities, such as having the capacity to learn from mistakes, being prepared for risks, and generally acting proactively in everyday work situations. Other facilitating skills were the capacity to act as a role model for colleagues and having experience from different areas of health care.

*You can lead by example. […] Try to comply with all the rules. You cannot correct others if they are not behaving or doing this or that unless you do it properly yourself.* N 6

Conversely, insufficient skills could act as a barrier to enhanced patient safety. Being a newly graduated nurse was mentioned as a barrier, due to a general lack of experience and the need for learning concerning many new aspects of work.

Those things that come more natural for the rest of us are unnatural for them [newly graduate nurse] and they barely have much time left [for the normal duties]. N 6

The nurses believed that feelings such as anxiety or worry about various aspects of their work could inhibit patient safety. They underscored that feelings of shame could lead to reluctance to admit to faults and reporting of incidents.

*It’s not always easy to report, because you feel ashamed and don’t want to confess that you have done something wrong or almost did something wrong.* N 12

The nurses perceived that the general fallibility of humans, for instance, insufficient attention to or awareness of various risks, could have a negative impact on patient safety. A lack of vigilance and reliance on habits could lead to reduced ability to detect potential problems.

*What do we have to be aware of? That we are humans and we make mistakes.* N 7

*I think you fall back on old routines and do what you have always done. Then you might handle things a bit carelessly. You send samples to the lab with the wrong name and so on. It’s not common, but it does happen.* N 11

### Team factors

Collaboration in multiprofessional teams was described by many nurses as an important factor for improved patient safety, whereas some nurses believed a lack of collaboration between physicians and other health care personnel functioned as a barrier to improved patient safety due to perceived hierarchical differences.

*We don’t work much in teams, which affects patient safety. I have worked in another hospital for 13 years. I experienced less hierarchy there than here.* N 3

*We do have a very close cooperation across the boundaries, so that physicians, nurses and the nurse assistants work relatively close together. And that, I believe, is an important part in ensuring that it is safe for patients.* N 8

Communication with colleagues in the workplace could influence patient safety positively or negatively, according to the nurses. The nurses believed that communication could facilitate patient safety when there was time set aside to discuss patients, there was an open climate that encouraged people to voice their opinion, and learning was encouraged.

*When you make a mistake, it’s important that you share this with others, so that you can learn […]We learn continuously and we, the staff, try to learn from what we do and don’t do and share with others.* N 12

However, ineffective communication could inhibit patient safety. The nurses mentioned that unwillingness to share knowledge could provide a barrier to improved safety.

*It sometimes feels like people prefer to keep things to themselves, ‘I won’t let anyone in on this.’* N 2

The nurses also described various disturbances in communication, for instance cell phones and various social media, which sometimes demanded their attention, resulting in ineffective transfer of information and knowledge. “Lost” information sometimes caused problems.

*We call it the whispering game. Sometimes it’s, ‘Yes, Anna told me this, but I misunderstood it because she was unclear.’ It can be really hard with communication. I might think I have been really clear and said that; ‘the daughter perceived it like this,’ but she might hear something different.* N 10

### Task and technology factors

The process of collecting, storing, and sharing patient safety-related data was identified by the nurses as an important facilitator for patient safety because it made risk factors more visible, thus promoting preventive efforts.

*I think that the quality registry [Senior Alert] has done a lot, because it makes it possible to detect risks early.* N 5

Senior Alert is a quality registry which documents risks regarding nutrition, falls and pressure ulcers, and specifies what measures have been or will be taken to minimize these risks. The nurses mentioned templates that required certain details to be filled in when entering information into the medical records. They believed these forms facilitate patient safety because they contribute to increased awareness of risks by highlighting critical areas.

*Well, at least three of these [keywords in templates] pop up automatically when I’m entering information about a patient [in the medical record]. And that, I believe, is also patient safety work.* N 2

On the other hand, medical records could also inhibit patient safety when they were not properly updated, contained outdated prescription information, lacked vital information, or included notes that were difficult to understand.

*If the information was clearer in the medical record, to make it explicit what we should do, then we wouldn’t have to do unnecessary things. That’s not good for the patient, either.* N 3

*For about two or three years ago we introduced computer-based medication lists. And I perceive at least, that the physicians often don’t know how they should prescribe so that we nurses are able to see in a clear way what to give and when and how.* N 11

Incident reporting was identified by the nurses as an important tool for enhanced patient safety. Feedback from incident reports raised awareness, fostered further reporting, and led to initiatives for improved care, especially when several reports pointed in the same direction or patterns could be detected. However, these systems also had their weaknesses, some of which might inhibit patient safety. Many nurses commented that not all incidents or risks were reported even though incident reporting had improved considerably. Some nurses remarked that the systems failed to capture small disturbances (such as lack of available wound care applies or a broken printer) that occurred in the workplace that might affect patient safety. The nurses also believed that insufficient data collection and poor dissemination of results, both formal and informal means of reporting, provided barriers to enhanced patient safety.

*You learn from these [incident reports] when you sit down and talk about it. You develop [by learning] from your mistakes all the time. That’s how you learn in patient safety, when something awful has occurred. At least it means it will never happen again.* N 10

Most nurses agreed that the use of computerized technology facilitated reporting and sharing of patient safety-related data and information, thus enhancing patient safety.

*This computer system that we now have here makes it easier to find information, I think, and that is also part of patient safety. It becomes easier; it is more transparent and visible.* N 8

However, many also complained about functionality issues, such as non-compatible systems, which they believed could inhibit ambitions for improved patient safety.

*You can’t answer the phone at the same time because if you interrupt yourself [in the computer system], it’s likely that you won’t be able to get back to the right place [in the computer program][…] You’ll lose the information.* N 1

The nurses believed that the availability and use of written protocols that provide structure for their work, such as guidelines and standardized care plans, positively influence patient safety. They mentioned that various forms of protocols saved time and provided guidance on what to do in specific situations, thus contributing positively to patient safety. However, many nurses emphasized the importance of using protocols as long as it did not inhibit their own critical thinking. Rather, they felt protocols must raise awareness and improve understanding. The nurses mentioned several examples of guidelines that were beneficial for patient safety, including hygiene and nutrition routines and various handbooks.

*I think the ‘The Handbook for Healthcare’ [title of a book with evidence based guidelines and instructions concerning patient care/treatments/examinations etc.] is very good, as it provides information on procedures about everything, really. You cannot just rely on what the person you’ve asked said. Where did that person get that information from? It probably works, but a reference is preferable.* N 11

### Work environment factors

Structures and forums for learning from errors were perceived as an important workplace factor that influenced patient safety. The nurses described several learning methods for influencing patient safety, including root cause analysis, quality registers, studies at their own work place, and continuous professional education on patient safety issues.

*I'm learning all the time. I feel that my colleagues are too […] We meet and discuss, and then I try to disseminate to others.* N 7

The nurses believed their work schedule influenced patient safety. They described that shift work, with irregular working hours, as something that inhibited patient safety because it led to non-continuity of care, both for the nurses who have to report to many different colleagues and for the patients who meet many different health care professionals.

*If I work one week on the ward, it may well be that I’m in three different teams during this week. And that’s not safe.* N 10

Staffing levels and the mix of competences also affected patient safety, according to the nurses. Patient safety was facilitated when there were a sufficient number of health care providers and the staff included a mix of competent and experienced providers. Conversely, one nurse said that locum physicians tended to have insufficient insight into the day-to-day-routines and usually lacked the mandate to initiate activities for improved patient safety, and that patient safety was compromised when the staff was stretched thin and/or there was a shortage of nurses with the right competences.

*This clinic is so specialized that it’s difficult to have a locum nurse here. It’s very hard. It’s almost impossible to replace a nurse here.* N 7

Various aspects of the physical environment were also perceived to influence patient safety. The nurses pointed out that patient safety was compromised when they worked in busy, hectic environments and when they were interrupted by telephones, for example, when they were handling medication. They believed disorganized environments and substandard or outdated equipment posed threats to patient safety.

*We need to buy new equipment. We have wheelchairs that are from the wartime period; they are about to fall apart. Things like that also play a part in patient safety work.* N 8

### Organizational and management factors

The nurses perceived leadership as an important factor that could both facilitate and inhibit patient safety. They observed that engaged managers provided a positive influence as they worked with the staff and learned what their strengths and weaknesses were. Conversely, many nurses pointed out that managers who had a negative attitude to patient safety-related issues or perhaps professed that patient safety was important but did not live up to their words could provide barriers to patient safety.

*Well, as long as the managers at the highest level don’t think it’s important nothing happens. It’s politically correct to say [that patient safety is important], but if they don’t really demonstrate [with action] that it’s important, well then we are getting nowhere.* N 3

Limited financial resources were mentioned by many nurses as an important barrier at the organizational level to achieving improved patient safety.

*That is something that you almost don’t say because it’s so obvious. You know that’s how it is, of course. That is probably the most important thing, having the resources to have fewer patients.* N 10

Restricted resources affected patient safety in many ways, according to the nurses. They said that there was not enough time or funding for ambitious patient safety work, including training in patient safety-related issues. Scarce resources also made it more difficult to address problems detected in risk analyses, medical records, or quality registries.

*I know that all patients that lie in bed need one of these extra mattresses. We have only two mattresses but four [patients] need them, but they haven’t been purchased because there is no money. So it’s a bit of an uphill struggle.* N 10

### Institutional context factors

The nurses believed that patient safety was affected by the availability and use of knowledge from external sources, such as health care providers employed in other areas of the health care organization or even outside their own health care organization. Some nurses described how the challenges of getting specialists from other clinics to see critically ill patients could jeopardize patient safety.

*When we get very ill patients, it is hard to get an on-call physician from a different clinic [specialty], such as surgery or medicine, because they have too much to do. And we have trouble getting our patients into intensive care [unit]; even though they have a bed available, they are still to be cared for in our ward. It’s a huge problem.* N 8

The nurses mentioned communication with people external to the workplace in negative terms regarding patient safety. They felt that reporting in transition of care was insufficient, for example, when transferring patients from municipal care to hospital care, and could inhibit patient safety.

*Many patients are admitted to hospital from municipal [care] and we often receive very little information concerning the patient. We usually have to make contact by telephone, but these patients are usually admitted to the hospital very quickly and are critically ill.* N 8

One nurse mentioned that communication problems between staff in different departments played a part in the death of one patient.

*The communication didn’t work; he [the patient] actually died. This [communication problem] was not the primary cause, but it probably contributed.* N 3

Societal interest in patient safety was thought to be important for prioritizing patient safety issues, such as infections.

*There are statistics to show [the magnitude of the problem]. That’s why I think an invasion of bacteria would put infection on the map [and lead to activity].* N 2

## Discussion

This study sought to explore important factors for patient safety, as perceived by registered nurses in general hospital care in Sweden. The nurses identified 22 factors that influenced patient safety, of which 12 functioned as both facilitators and barriers, six constituted barriers, and four were facilitators. These 22 factors belonged to many different levels, ranging from patient interaction and individual staff to institutional context. There were no specific patterns in which barriers or facilitators were not more common at any level. They were quite evenly distributed at the different levels.

Our findings indicate that the nurses had a multilevel perspective of patient safety, viewing facilitators and barriers to patient safety in terms of several system levels. Nurses often have an important role as coordinators of multidisciplinary care and are involved in many aspects of patient care. Their role facilitates systems thinking because they are able to see interrelationships, patterns, and underlying structures that reveal the dynamic interplay among system components. The importance of systems thinking in understanding the dynamics of the big picture and the multiple levels of interacting influences on patient safety has been emphasized since the publication of the Institute of Medicine report *To Err is Human* in 1999 [21-23].

The multifaceted systemic nature of patient safety facilitators and barriers suggests that solutions will need to be as complex as the problem, targeting different levels such as individual health care practitioners, teams, departments, and organizations [24]. In Sweden, the Swedish Association of Local Authorities and Regions (SALAR) has taken a multifaceted approach to patient safety by promoting a multitude of initiatives [25]. Although many of these efforts and activities have considerable face validity, solid evidence of effectiveness is lacking for many patient safety solutions because they are often too complex to study under rigorous controlled conditions. There is debate among patient safety researchers and policy makers regarding the degree to which various solutions should or could be studied scientifically before dissemination and implementation [5,7,15].

The nurses mentioned several factors that are associated with patient safety culture, including collaboration in multiprofessional teams, communication with colleagues, leadership, and structures and forums for learning from errors. Patient safety culture, which is one aspect of the wider culture of the organization, has been defined as the shared assumptions, values, and norms among members of an organization or group concerning practices that directly or indirectly influence patient safety [26,27]. The Institute of Medicine report recommended that health care organizations create an environment in which safety culture is an explicit organizational goal and a leadership-driven top priority [1]*.* There is emerging evidence to support the potential effectiveness of solutions to promote improved safety culture. The best evidence to date seems to include solutions comprising multiple components that incorporate team training and mechanisms to support team communication, and include management engagement in front-line safety work [28-31].

The culture of an organization is often studied as an organization-wide issue, but researchers have increasingly shown that subcultures should be studied to develop an in-depth understanding of an organization’s culture [32,33]. A subculture in an organization is “a group or unit in an organization that is in frequent interaction perceives itself to be distinct from other groups in the organization, and that shares similar problems as well as in-group understanding of ways of solving such problems” [34]. An important issue is the extent to which the nurses’ views on facilitators and barriers to patient safety overlap with factors identified by other professions. Studies have shown that the perception of patient safety and how it can be improved differs depending on the position in the health care system [35-37] and the profession [38,39]. There is a need for studies of other professions to investigate whether the subcultures are aligned with the organization-wide safety goals and the extent to which there are interprofessional communication and collaboration difficulties that affect patient safety.

Communication is closely linked with hierarchy, with research showing that the authority gradient, i.e. the psychological distance between individuals and professional groups, can lead to withheld information or information being adapted to suit the recipient. Commercial airline crews have learned to speak up and raise concerns, contributing to a remarkable safety record in commercial aviation over the past 50 years [15]. While some nurses in our study perceived hierarchical difference between nurses and physicians as a barrier to patient safety, research by Berbyuk *et al*. [40] suggests that the professional hierarchy in Swedish health care is rather flat and that the authority gradient is relatively low [40]. The lack of steep hierarchies could mean that most nurses feel comfortable raising their concerns with physicians, but it could lead to unclear professional roles which would cause other difficulties. Learning is widely considered to be a critical aspect of a beneficial safety culture [7,41]. The nurses in our study pointed to several issues related to learning, including the importance of having time and structures for discussions on patient safety issues and the skills, abilities, and interest that may affect patient safety. Organizations learn through their individual members, yet organizational learning is not simply the sum of the learning of each of its members. Organizational learning is usually conceived as a process that results in relatively lasting changes in organizational practices due to changes in routines, rules, norms, strategies, and technologies that are assumed to guide the behavior of the organization’s members [42,43]. A learning organization is competent in creating, acquiring and transferring knowledge, and modifying its behavior to reflect new knowledge [44]. However, the nurses in our study mentioned disturbances in communications with their colleagues and with patients, and they noted that all incidents were not reported and that feedback was not always given for reported incidents, thus suggesting that organizational learning was difficult.

Feedback is also imperative to achieve continuous high rates of incident reports [45,46]. Incident reporting was mentioned by the nurses as important for patient safety, which was expected considering that local reporting systems have been given a dominant role in efforts to improve patient safety in Sweden [47]. However, the reliance on incident reporting systems in many countries has been criticized because these systems are insufficient on their own to identify incidents [48,49]. The systems have also been described as being too outcome oriented, resulting in an incomplete picture of the event [50]. The nurses in our study group emphasized the importance of having meetings where they can discuss and reflect on the events for increased learning.

The nurses believed patient interaction and engagement were important for patient safety. There is an international trend towards greater patient involvement in health care delivery. Government policies in many countries aim for patients to be generally more involved in their health care [51]. However, despite increased international emphasis on patient involvement in safety, there is a paucity of research findings on the acceptability to patients of a new patient role and the extent to which such involvement actually leads to safety improvements [52]. Research has identified numerous barriers to enlisting patients in efforts to improve patient safety, including limited acceptance of a more active patient role [53] and insufficient health literacy, that is, the capacity to obtain, process, and understand basic health information and the services needed to make appropriate health decisions [54]. One facilitator, as our nurses also emphasized, is that the patient’s relatives should be included and involved in the care, if acceptable to the patient [55,56]. There have been calls for more research for better understanding of how patients can be involved in their own care [51,52].

We have found few comparable studies on hospital nurses’ perception of factors of relevance for patient safety. Other studies within this field have either been set in a specific setting such as the operating theatre or intensive care [57-59], examined quality improvement work [22,60,61], been limited to one factor such as communication or medical errors [45,46,62], or investigated the perspectives of other professional groups [13]. However, two studies are broadly similar to our study. A study by Currie and Watterson [60] set in the United Kingdom focused mainly on barriers and quality improvement, identifying training and education in patient safety-related issues as essential to achieve improved patient safety. They also mentioned organizational context and organization and management of care as important factors, which were also mentioned by nurses in our study. A Canadian study by Nicklin and McVeety [63] emphasized the importance of work environment–related factors, including issues related to workload, nursing shortages, and the physical environment. These somewhat overlapping results might suggest that other health care systems in other countries present similar challenges as those in Swedish health care. Further research on patient safety perceptions of nurses and other professional groups is warranted. It is also important to analyze these perceptions in relation to the existing evidence base for various solutions.

The researchers behind this study have previously undertaken a survey of patient safety officers in the county councils, i.e. the meso level of Swedish health care, to identify factors they believed to be of relevance for improved patient safety [13]. There was some overlap with the findings of this micro-level study, including the importance attributed to patient safety culture and communication. The previous study also pointed to the importance of education and training in patient safety issues. Considering the paucity of evidence-based patient safety practices, it is important to investigate perceived facilitators and barriers for improved patient safety at different levels of health care since the “patient safety determinants” do not necessarily correspond.

This study has a number of shortcomings that must be considered when interpreting the results. The sample size was relatively small (12 nurses) although the data analysis confirmed that data saturation was reached before cessation of the interviews. The voluntary nature of participation in the study means that the nurses interviewed may differ from the broader population of nurses. These nurses were recommended by patient safety officers in their county council and almost half of them have an explicit patient safety responsibility in their duty. This could bias the results to some extent since they are likely to be interested in patient safety issues and might have more insights into factors that contribute to or detract from improved patient safety. However, knowledgeable interviewees have probably given us a fuller interview material, and there is little evidence that imply other, less interested, nurses would mention other factors.

The factors associated with patient safety are not intended as an exhaustive list of all possible facilitators and barriers to patient safety as perceived by nurses; other studies may yield partially different factors or give different priorities to various factors. The interviews were mostly conducted by telephone. Although face-to-face interviews have the advantage of eliciting non-verbal information, telephone interviews allow the researcher to follow and respond to the interviewee through language and reflection, which has the potential of making the interviewee’s descriptions richer [64]. Telephone interviews were also practical as the participants were from different areas in Sweden. They were suitable for the descriptive aim of the study [60]. Further, research suggests that transcriptions of telephone and face-to-face interviews do not differ much [14]. The telephone interviews were shorter in duration, but we did not notice any differences with regard to the richness of the interview material.

The study also has strengths. The decision to conduct interviews instead of undertaking a survey was based on the belief that the interviews would lead to a more in-depth understanding of the factors concerning patient safety that are important to front-line nurses. The multidisciplinary research team may also be considered a strength, permitting different perspectives on the issues under study. The team consisted of a nurse (MR) with experience from clinical patient work and work with safety issues such as conducting risk- and root cause analyses, a researcher (KR) with a background in implementation science, innovation research and medical technology research and a researcher (PN) with experience from implementation science and injury prevention research. We believe the study has provided unique information on factors contributing to improved patient safety according to nurses in Sweden.

### Implications

Nurses play a vital role in patient safety. We believe this study will be useful as a basis for discussions on how improved patient safety can be achieved among health care personnel at all levels of health care, from those in training to front-line staff and senior managers, as well as participants in the patient safety network coordinated by SALAR. Although this study focused on nurses’ perceptions, other professional groups may echo some of the facilitators and barriers to improved patient safety identified here, but there may also be differences that warrant further investigation.

The study points to the relevance of a multifaceted system perspective on patient safety problems and solutions. The results imply that improvements are possible in the area of communication, for example in the handoff of patients to another caregiver, i.e. transferring primary patient responsibility and essential information about a patient’s condition to oncoming staff. Communication is also an important part of patient safety culture, an area which has attracted an increased attention since the publication of the Institute of Medicine’s report and is an important focus of SALAR’s initiatives. Some of the findings show that nurses have positive attitudes to involving patients in their care, for potential safety benefits. However, this is an area where more research is needed.

## Conclusions

A broad range of factors are important for patient safety according to registered nurses in general hospital care in Sweden. The nurses identified facilitators and barriers to improved patient safety at multiple system levels, indicating that complex multifaceted initiatives are required to address patient safety issues. This study encourages further research to achieve a more explicit understanding of the problems and solutions to patient safety.

## Competing interests

This work was supported financially by the Swedish Association of Local Authorities and Regions (SALAR). The authors report no conflicts of interest. The authors alone are responsible for the content and writing of the paper.

## Authors’ contributions

MN, PN, and KR designed the study; MN collected the data; MN, PN and KR analyzed and interpreted the data, MN and PN drafted the manuscript; PN and KR critically revised the manuscript. All authors read and approved the final manuscript.

## Pre-publication history

The pre-publication history for this paper can be accessed here:

http://www.biomedcentral.com/1472-6955/13/23/prepub
